# Docking site complications analysis of Ilizarov bone transport technique in the treatment of tibial bone defects

**DOI:** 10.1186/s13018-023-04356-6

**Published:** 2023-11-22

**Authors:** Dongwei Feng, Yaxin Zhang, Weize Wu, Heping Jia, Chuang Ma

**Affiliations:** 1 Department of Pain, The First Affiliated Hospital of Hebei North University, Zhang Jiakou, Hebei China; 2 International Medical Services, The First Affiliated Hospital of Hebei North University, Zhang Jiakou, Hebei China; 3 Department of Joint Surgery, The First Affiliated Hospital of Hebei North University, Zhang Jiakou, Hebei China; 4 Department of Orthopaedic, The First Affiliated Hospital of Xinjiang Medical University, Urumqi, Xinjiang China

**Keywords:** Bone transport, Ilizarov technique, Complication, Tibia, Docking site

## Abstract

**Background:**

Treating long bone defects of the extremities caused by trauma, infection, tumours, and nonunion has been challenging for clinical orthopaedic surgeons. Bone transport techniques have the potential to treat bone defects. However, inevitable docking site complications related to bone transport techniques have been reported in many studies. The purpose of this study was to investigate the risk factors associated with docking site complications in patients who underwent the Ilizarov bone transport technique for the treatment of tibial bone defects.

**Methods:**

This retrospective study included 103 patients who underwent bone transport for the treatment of large bone defects in the tibia from October 2012 to October 2019. Patient demographic data, complications and clinical outcomes after a minimum of 2 years of follow-up were collected and retrospectively analysed. Additionally, univariate analysis and logistic regression analysis were used to analyse the factors that may affect the development of docking site complications in patients with tibial bone defects treated with the Ilizarov bone transport technique. The clinical outcomes were evaluated using the Association for the Study and Application of the Ilizarov criteria (ASAMI) at the last clinical follow-up.

**Results:**

All 103 patients with an average follow-up of 27.5 months. The docking site complications rate per patient was 0.53, and delayed union occurred in 22 cases (21.4%), axial deviation occurred in 19 cases (18.4%) and soft tissue incarceration occurred in 10 cases (9.7%). According to the results of the logistic regression analysis, the bone defect length (*P* = 0.001, *OR* = 1.976), and bone defect of distal 1/3 (*P* = 0.01, *OR* = 1.976) were significantly correlated with delayed union. Bone defect length (*P* < 0.001, *OR* = 1.981) and external fixation time (*P* = 0.012, *OR* = 1.017) were significantly correlated with axial deviation. Soft tissue defects (*P* = 0.047, *OR* = 6.766) and the number of previous operations (*P* = 0.001, *OR* = 2.920) were significantly correlated with soft tissue incarceration. The ASAMI bone score at the last follow-up showed a rate of excellent and good bone results of 95.1% and a rate of excellent functional results of 90.3%.

**Conclusion:**

The Ilizarov bone transport technique is a practical and effective method for the treatment of tibial bone defects. However, the incidence of complications at the docking site is high, of which bone defect length, external fixation time, the number of previous operations, soft tissue defects and the bone defect of distal 1/3 are statistically significantly associated with the occurrence of docking site complications.

## Introduction

The treatment of long bone defects of the extremities caused by acute trauma, posttraumatic sequelae, resection due to tumour or bone infection, nonunion or congenital deficiencies has been a challenge for clinical orthopaedic surgeons and patients [1]. Although traditional techniques such as acute shortening, autogenous bone grafts, free vascularized fibular transfer and the Masquelet technique have been somewhat effective for treating long bone defects of the extremities, each has significant limitations [2]. The bone transport (BT) technique proposed by Professor Ilizarov in 1989 is a method to repair bone and soft tissue defects using external fixation techniques that has successfully saved many limbs on the verge of amputation [7]. The Ilizarov fixator addresses not only the problem of the bone defect but also any malalignment, shortening, or soft-tissue loss [8]. It is unfortunately associated with a multitude of docking site complications [9].

There are few studies focusing on the risk factors for docking site complications in patients with tibial bone defects treated with the Ilizarov technique. The purpose of this study was to identify and analyse the causes and risk factors associated with docking site complications in patients with tibial bone defects treated with the Ilizarov technique.

## Methods

This study was approved by the Ethics Committee of our institution. Informed written consent was obtained from the participants. In addition, this study was performed in line with the international ethical guidelines for studies involving human subjects according to the Declaration of Helsinki.

### Patients

There are115 cases of tibial bone defects treated by the Ilizarov bone transport technique from October 2012 to October 2019 were included in our study. Inclusion criteria: [1]. The age of the patients was between 17 and 65 years old [2]. The length of the tibial bone defect ≥ 3 cm; [3]. The follow-up period was longer than 24 months. Exclusion criteria: [1]. Patients with systemic diseases, such as liver and kidney insufficiency, bone metabolism dysfunction and other related diseases [2]. Patients with a nerve or blood vessel injury or disease of the affected limb [3]. Patients with poor compliance or who were unable to cooperate with treatment and follow-up.

During the study period, 115 patients who were treated for tibial bone loss using Ilizarov bone transport technique were identified. After application of the exclusion criteria, 103 patients were included in the study. There were 90 males and 13 females with a mean age of 37 years (range 17–66 years). The aetiology was traumatic bone loss in 25 patients, osteomyelitis in 61 and nonunion in 17. There were 19 cases in the proximal 1/3 of the diaphysis, 39 in the middle 1/3 and 45 in the distal 1/3. There were 12 limbs with active infections with sinus and drainage. Seventeen patients suffered from soft tissue defects after debridement. The mean bone defect size was 6.6 cm (range 3–13 cm). Single bone transport was performed in 80 patients, and double bone transport was performed in 23 patients.

### Surgical technique

The surgical procedure was planned according to standard AP(anteroposterior) and lateral radiographs of the affected limb. The relevant examination was conducted, surgical contraindications were assessed, and the wound was thoroughly debrided under general anaesthesia or epidural anaesthesia. Prior to bone transport, all hardware were removed, all necrotic and infected bone and soft tissue were subjected to radical debridement, and an antibiotic-impregnated cement spacer was implanted, if necessary, to improve stability. For infected persons, surface secretions and deep scraped tissues were retained for bacterial culture and drug sensitivity tests to guide follow-up anti-infection treatment. Cortical bleeding, described as the so-called paprika sign [10], was accepted as an indication of vital osseous tissue. A local tissue flap or direct tension-free suture was applied to reconstruct the small soft tissue defects, whereas flap transfer or free skin grafting was used to cover the larger wound.

Bone transport was initiated when clinical manifestations and laboratory indicators showed that the infection had resolved. Preoperative anteroposterior and lateral X-rays were used to evaluate the defect size and plan the construction of the external fixator. The type of external fixator was comprehensively determined by the location of the bone and soft tissue defect along with the surgeon’s experience and patient’s preference. The osteotomy was performed in a minimally invasive fashion using the Gigli saw technique, and special care was given to preserve as much periosteum as possible. Bone defects larger than 8 cm or exceeding 40% of the injured bone underwent a double-level bone transport procedure [11]. All procedures were conducted by the same surgical team.

### Postoperative management and follow-up

Regular pin-site care. All patients were encouraged to perform isometric muscle and joint range of motion (ROM) exercises within an acceptable range of pain tolerance on the second day after surgery. Antibiotics that are suitable according to the results of cultures and antibiotic susceptibility tests are applied intravenously for at least 3 weeks or until the erythrocyte sedimentation rate (ESR) and C-reactive protein (CRP) levels return to within normal limits.

After a latency period of 7–10 days, bone transport was started at a rate of 1 mm (single level) or 2 mm (double level) daily, 4 times a day. The rate of bone transport was adjusted according to the patients’ tolerance and the quality of the regeneration. The bone transport procedure was continued for 4 or 5 days to compress the docking site after the docking. The external fixator was dynamized before removal. The external fixator was removed when the standard orthogonal radiographs showed sufficient consolidation of the distraction zone (dense bone formation) and solid docking site union (corticalization in 3 of 4 cortices)[12]. Additionally, all patients were placed on a functional brace for 4–6 weeks to protect against refracture.

### Data collection

Demographic and clinical data were collected, including sex, age, number of previous operations, type of external fixation (circular (TrueLok Ring Fixation System, Orthofix, Verona, Italy) or monolateral (Limb Reconstruction System, LRS, Orthofix, Verona, Italy)), distraction regenerate length (DRL), docking time (DT), external fixation time (EFT), external fixation index (EFI) and type of difficulties that occurred during and after the bone transport procedure. The EFT referred to the time spent before removal of the external fixator. The EFI was defined as the ratio of the days of EFT to the DRL (centimetres). Radiographic evaluation was conducted every 2 weeks during the bone transport period and monthly in the consolidation phase. All patients were closely followed up at a minimum of 2 years after the removal of the external fixator.

Complications were classified according to the criteria in Paley et al. [13]. All complications were divided into minor and major complications. Minor complications did not affect the final result or required nonoperative or a minor operative intervention, while major complications required a more complex and unplanned operative intervention or resulted in permanent sequelae. Bony and functional outcomes were assessed at the last follow-up using the ASAMI score [14].

### Statistical analysis

Continuous variables (age, bone defect length, number of previous operations, etc.) were compared by using t tests, and Pearson’s chi-square test or Fisher’s exact test was used to compare categorical variables (sex, type of external fixation, soft tissue defect, location of bone defect and single double level). The variables with significant differences, as indicated by a *p value* < 0.05 in the univariate analysis, were brought into the binary logistic regression analysis for analysis of related risk factors; the results with a *p value* < 0.05 had differences. SPSS version 22.0 (IBM Corp, USA) was used to analyse all data.

## Result

All patients were followed up for an average of 27.5 (24–48) months after removal of the external fixator, the soft tissue was successfully managed by musculocutaneous flap transfer in 17 cases, and all patients achieved bone healing. The mean EFT was 293.30d (176–473d), the mean EFI was 53.33d (36.73–77.56d), and the mean DT was 72.3d 46–68d). Based on the ASAMI bone score, the bony result was excellent in 91 patients, good in 7, fair in 3 and poor in 2. The ASAMI functional result was excellent in 67 patients, good in 26, fair in 8, and poor in 2. The details are shown in Table 1.

**Table 1:** Bone transport-related complications

Complication	Minor	Major
Pin-site infection	60	1
Axial deviation	12	7
Delayed union	2	20
Soft tissue incarceration	2	8
Joint stiffness	4	7
Ankle stiffness	7	10
Muscle contractures	6	2
Nonuion	0	4
Refracture	0	0
Nerve damage	0	0
Vascular trauma	0	0
Total	93	59

Complications were classified according to Paley classification, and, a total of 55 complications occurred in the docking site with an average of 0.53 complications per patient (16minor complications and 39major complications). No case encountered vascular or nerve compromise. Delayed union at the docking site occurred in 22 patients (21.4%), 20 of them were treated with compression with an external fixator after bone grafting, and the remaining patients achieved union after compression with an external fixator. Axial deviation occurred in 19 patients (18.4%) among which 7 cases were deviated greater than 5°, for recurvature purposes, modification of the apparatus or inserting an additional Schanz screw(s) to pull the bone out of its deviated position was required before the end of the treatment. Soft tissue incarceration was encountered 10 cases (9.7%). After the ends of the bone were freshened, the medullary canal was opened, the interposed soft tissue was excised, and the iliac bone graft was introduced; the details are shown in Table 1.

Among the univariate variables, there was a significant difference between the delayed union group and the nondelayed union group in terms of clinical bone defect length (8.46 ± 2.09 > 6.04 ± 1.89, *P* < 0.001), EFT (318.72 ± 67.54 > 286.40 ± 52.94, *P* = 0.003), EFI (59.72 ± 9.28 > 51.60 ± 8.86, *P* < 0.001), and bone defects of distal 1/3 (35.56% > 18.08, *P* = 0.007). In contrast, there was no significant difference between the two groups in terms of the remaining variables. Logistic regression analysis showed that the length of the bone defect and the presence of the defect in the distal 1/3 of the bone were risk factors for delayed union, and the *OR* values were 1.976 and 11.379, respectively. The details are shown in Tables 2 and 3.

**Table 2:** Comparison of delayed union / non-delayed union

Demographic date	Delayed union	Non-delayed union	t/${x}^{2}$	*P*
*Sex*				
Male	18 (26.32)	72 (73.68)	0.784	0.376
Female	4 (17.86)	9 (82.14)		
*Type of external fixation*				
monolateral	19 (20.65)	73 (79.35)	0.256	0.613
circular	3 (27.27)	8 (72.73)		
*Soft tissue defect*				
yes	6 (35.29)	11 (64.71)	2.354	0.125
no	16 (18.60)	70 (81.40)		
*Level of bone transport*				
Single	16 (20.00)	64 (80.00)	0.394	0.530
Double	6 (26.09)	17 (73.91)		
*Location of bone defect*				
proximal 1/3	1 (5.26)	18 (94.74)	10.023	0.007
middle 1/3	5 (12.82)	34 (87.18)		
distal 1/3	16 (35.56)	29 (64.44)		
Age	42.27 ± 14.66	36.54 ± 13.10	− 1.773	0.079
Previous operation time	4.00 ± 1.45	3.54 ± 1.11	− 1.602	0.112
Size of bone defect	8.46 ± 2.09	6.04 ± 1.89	− 5.188	< 0.001
DT	76.77 ± 12.72	71.09 ± 13.09	− 1.818	0.072
EFT	318.72 ± 67.54	286.40 ± 52.94	− 2.389	0.003
EFI	59.72 ± 9.28	51.60 ± 8.86	− 3.772	< 0.001

**Table 3:** Risk factors of delayed union

Variables	β	Standard deviation	Statistical value	P value	OR value	95%CI
Size of bone defect	0.681	0.198	11.837	0.001	1.976	1.340–2.912
EFI	0.041	0.037	1.232	0.267	1.042	0.969–1.120
EFT	0.003	0.006	0.338	0.561	1.003	0.992–1.015
*Location of bone defect*						
Proximal 1/3			5.860	0.053		
Middle 1/3	0.877	1.247	0.495	0.482	2.404	0.209–27.706
Distal 1/3	2.432	1.201	4.098	0.043	11.379	1.080–119.850

Among the univariate variables, there was a significant difference between the axial deviation group and the nonaxial deviation group in terms of clinical bone defect length (8.74 ± 1.45 > 6.07 ± 1.99, *P* < 0.001), EFT (340.95 ± 80.25 > 282.52 ± 45.16, *P* = 0.006), and EFI (59.66 ± 10.77 > 51.90 ± 8.65, *P* = 0.001). In contrast, there was no significant difference between the two groups in terms of the remaining variables. Logistic regression analysis showed that bone defect length and EFT were risk factors for axial deviation, and the *OR* values were 1.981 and 1.017, respectively. The details are shown in Tables 4 and 5.

**Table 4:** Comparison of axial deviation /non- axial deviation group

Demographic date	Axial deviation	Non-axial deviation	t/${x}^{2}$	*P*
*Sex*				
Male	17 (18.89)	73 (81.11)	0.093	0.761
Female	2 (46.43)	11 (53.57)		
*Type of external fixation*				
Monoliteral	18 (19.57)	74 (80.43)	0.717	0.397
Circular	1 (9.09)	10 (90.91)		
*Soft tissue defect*				
Yes	3 (17.65)	14 (82.35)	0.009	0.926
No	16 (42.94)	70 (57.06)		
*Level of bone transport*				
Single	15 (18.75)	65 (81.25)	0.022	0.882
Double	4 (17.39)	19 (82.61)		
*Location of bone defect*				
Proximal 1/3	2 (10.53)	17 (89.47)	2.384	0.304
Middle 1/3	10 (25.64)	29 (74.36)		
Distal 1/3	7 (15.56)	38 (84.44)		
Age	39.16 ± 14.16	37.42 ± 13.57	− 0.501	0.617
Previous operation time	4.21 ± 1.08	3.51 ± 1.19	− 2.351	0.021
Size of bone defect	8.737 ± 1.45	6.07 ± 1.99	− 5.512	< 0.001
DT	77.21 ± 10.58	71.19 ± 13.48	− 1.822	0.071
EFT	340.95 ± 80.25	282.52 ± 45.16	− 3.065	0.006
EFI	59.66 ± 10.77	51.90 ± 8.65	− 3.371	0.001

**Table 5:** Risk factors of axial deviation

Variables	β	Standard deviation	Statistical value	*P*	OR value	95% CI
Size of bone defect	0.683	0.185	13.646	< 0.001	1.981	1.378–2.846
Previous operation time	0.325	0.287	1.283	0.257	1.384	0.788–2.431
EFI	0.029	0.037	0.640	0.424	1.030	0.958–1.106
EFT	0.017	0.007	6.360	0.012	1.017	1.004–1.031

Among the univariate variables, there was a significant difference between the soft tissue incarceration group and the nonsoft tissue incarceration group in terms of clinical soft tissue defects (29.41% > 5.81%, *P* = 0.003), number of previous operations (5.50 ± 1.27 > 3.44 ± 1.00, *P* < 0.001), and EFT (339.70 ± 69.97 > 288.31 ± 54.19, *P* = 0.007). In contrast, there was no significant difference between the two groups in terms of the remaining variables. Logistic regression analysis showed that soft tissue defects and the number of previous operations were risk factors for soft tissue incarceration, and the OR values were 6.766 and 2.920, respectively. The details are shown in Tables 6 and 7.

**Table 6:** Comparison of soft tissue incarceration /non- soft tissue incarceration group

Demographic date	Soft tissue incarceration	Non-soft tissue incarceration	t/${x}^{2}$	P
*Sex*				
Male	9 (10.00)	81 (90.00)	0.069	0.793
Female	1 (7.69)	12 (92.31)		
*Type of external fixation*				
Monolateral	9 (97.83)	83 (2.17)	0.005	0.942
Circular	1 (9.09)	10 (90.91)		
*Soft tissue defect*				
Yes	5 (29.41)	12 (70.59)	9.017	0.003
No	5 (5.81)	81 (94.19)		
*Level of bone transport*				
Single	9 (11.25)	71 (88.75)	0.971	0.324
Double	1 (4.35)	22 (95.65)		
*Location of bone defect*				
Proximal 1/3	1 (5.26)	18 (94.74)	0.543	0.762
Middle 1/3	4 (10.26)	35 (89.74)		
Distal 1/3	5 (11.11)	40 (88.89)		
Age	37.90 ± 13.89	37.72 ± 13.62	− 0.032	0.974
Previous operation time	5.50 ± 1.27	3.44 ± 1.00	− 6.000	< 0.001
Size of bone defect	7.75 ± 2.66	6.43 ± 2.08	− 1.854	0.067
DT	77.90 ± 11.58	71.70 ± 13.23	− 1.423	0.158
EFT	339.70 ± 69.97	288.31 ± 54.19	− 2.768	0.007
EFI	60.98 ± 12.88	52.51 ± 8.78	− 2.030	0.07

**Table 7:** Risk factors of soft tissue incarceration

Variables	β	Standard deviation	Statistical value	*P* value	OR value	95% CI
Soft tissue defect	1.912	0.963	3.938	0.047	6.766	1.024–44.712
Previous operation time	1.072	0.324	10.930	0.001	2.920	1.547–5.512
EFT	0.012	0.007	2.992	0.084	1.012	0.998–1.026

## Discussion

Current treatments for bone defects include autologous or allogeneic bone grafts, vascularized free fibula grafts, Masquelet membrane induction techniques, and Ilizarov bone transport techniques [2]. Although autologous bone grafting is the gold standard for the treatment of bone defects, there is a potential for donor site-related complications, and Chimutengwende et al. [15] do not recommend its use in bone defects more than 5 cm. Allogeneic bone grafting can be performed using allogeneic bone from the same site as the bone defect, but there is a risk of rejection reaction and potential infection with infectious diseases. Borzunov et al. [16] do not recommend the use of allogeneic bone grafting for the treatment of long bone defects. A vascularized free fibula graft can be used to reconstruct bone defects larger than 6 cm, but its placement during microsurgery is technically demanding and carries the risk of stress fractures [17]. The Masquelet membrane induction technique has advantages in the management of infected bone defects, but long-term bed rest, restricted weight-bearing, and donor site-related complications limit the application of this technique [18]. With the improvement of external fixation devices and the development of microsurgical techniques, bone transport techniques based on the concept of “distraction osteogenesis” described by Ilizarov have been rapidly promoted at home and abroad because of their simplicity, minimal invasiveness, effectiveness and protective biomechanical environment required for bone healing [19].

However, a lengthy external fixation time and an overall increased risk for complications have become the main obstacles to overcome for its extended application. After prolonged transport for the treatment of long bone defects, the bone end at the site of nonunion is covered by fibrous or fibrocartilaginous tissue, and the medullary canal disappears, where it usually disappears soft tissue located between the bone ends [20]. Complications such as soft tissue incarceration, delayed union, nonunion, and axial deviation may occur at the docking site, and there is also a risk of refracture after removal of the external fixator. In this study, delayed union occurred in 22 cases, the incidence of axial deviation of the docking site, soft tissue incarceration and nonunion was 18.4% (19/103), 9.7% (10/103) and 3.9% (4/103), respectively, and the frequency of complications was 0.53 times/case, which was also similar to the study by Spiegl et al. [21] (frequency of complications was 0.64 times/case). The incidence of delayed union at the docking end was higher in this study (21.4%) than in previous studies [22]. This is because osteomyelitis involves a relatively large portion of bone and requires repeated debridement before the initiation of bone transport, and the microenvironment for bone regeneration and soft-tissue coverage may be destroyed. Union at the docking site becomes a time-consuming process. The goal of treatment is managing these patients in one stage with a shorter course of treatment and fewer docking site complications.

The tibia itself is physiologically curved, and bone transport involves mechanical linear motion, so there is often axial deviation during bone transport. This study shows that bone transport distance and EFT are risk factors for axial deviation. Aarnes et al. [23] similarly found that axial deviation were more likely to occur with increasing bone transport distance. The gastrocnemius muscle is mainly located in the posterolateral tibia, and with the increase in distraction distance, the bone segment often shows different degrees of deviation, which is caused by the presence of tension angulated from the alignment in the external fixation system. At the same time, osteoporosis and screw-bone reactions are more likely to occur with longer EFTs, resulting in decreased mechanical properties of the overall structure of the external fixator and the shaft [24]. In addition, axial deviation can occur due to insufficient contact area at the docking site. The principle of screw placement mode in bone transport segments is balanced screw placement ("near and far") [25]. However, there are differences in the aetiology and soft tissue conditions of the patients, which affect the placement angle and number of Schanz nails as well as full needles. Other factors that may influence axial deviation include age, osteotomy position, and biomechanical environment of different diaphyses.

The authors concluded that in the application of a monorail external fixator, increasing the number of Schanz nails can improve the mechanical stability of the bone transport segment, and hydroxyapatite-coated Schanz nails can potentially enhance the stability of pin-bone interface. At the same time, pin-wires should not be placed at the site of osteoporosis. When using a circular external fixator, the suitable tension provided by a Kirschner wire needle is 1200 N; a needle that provides inadequate tension will reduce the stability of the external fixator, and Kirschner wires that are too large will easily fracture. The proximal tibial external fixation component was positioned appropriately close to the medial and anterior proximal tibia. Apivatthakakul [26] and Liodakis [27] chose bone transport over an intramedullary nail and MIPPO to avoid axial deviation. However, it is debatable whether an intramedullary nail can be used in patients with osteomyelitis [28]. Barbarossa \* MERGEFORMAT [29] reported the rate of axial deviation was 34.3%, He achieved the improvement of the axis < 7 in five patients by correcting the frames and adding the wires in general or spinal anesthesia. The axial deviation is 18.4% in our study, which was lower than previous studies, This may be because we adjusted the external fixator in time for patients with axial deviation. Eventually, 7 patients with an angle of deviation > 5° underwent correction of the axial deviation by surgery or by the placement of new components. Regular and timely follow-up can effectively reduce the incidence of axial deviation.

The possible reasons for soft tissue incarceration occurrence may be subcutaneous structural abnormalities. This study shows that soft tissue defects and the number of previous operations were risk factors for soft tissue incarceration. The greater the number of previous operations, the more severe the soft tissue scar adhesion, and the tougher the texture between the soft tissue and the bone segments, the more the soft tissue accumulates at the segment ends as handling proceeds [30]. In addition, we found that soft tissue defects were more likely to have soft tissue incarceration. Similarly, Paley et al. [31] found in a retrospective analysis that all patients with soft tissue incarceration had undergone flap repair of the wound. The soft tissue after flap repair of the wound is thick and flaccid and will also accumulate at the bone ends as handling proceeds. Recently, Chen Hui et al. [32] retrospectively analysed 12 cases of tibial bone defects with soft tissue defects and avoided the occurrence of soft tissue incarceration by placing a tissue expander at the bone ends, but only a few cases were included, and its effectiveness needs to be further explored. Aihemaitijiang et al. [17] believes that the "accordion technique" can lyse and absorb the incarcerated tissue, but it requires the cooperation of the patient. Additionally, there were cases in which a wire was used instead of stitches to protect the skin from collapse according to a report in the literature, but the patient sustained considerable trauma to the body, which caused great inconvenience during wound dressing changes. In this study, 10 cases of the patients had soft tissue incarceration, revision at the docking site was undertaken as soon as the transported segment had reached the target site. Freshening the bone ends, opening the medullary canal, excising the interposed soft tissue, and introducing iliac bone grafts when necessary promote healing of the docking site.

As a result of the poor blood supply and the factors of contact surface deviation, delayed union of the docking site is also a common complication. This study shows that bone transport distance and distal 1/3 bone defects are risk factors for delayed union. However, a relatively weak blood supply to the distal 1/3 of the tibia can affect callus formation at the segmental end. However, there is physiological curvature in the distal 1/3 of the tibia, and malalignment often occurs at the docking site, which may be a more important factor causing delayed union [15]. Lavini et al. [33] found that the docking site gradually atrophies and loses its activity as the distraction distance increases, showing delayed union and even nonunion. After long transport for an extensive bone defect, the bone ends at the site of nonunion become covered with fibrous or fibrocartilaginous tissue. Related studies [34] have also found that smoking not only inhibits osteoblast production but that nicotine is also a vasoconstrictor and adversely affects bone end blood supply. Nonsteroidal anti-inflammatory drugs [35] also reduce osteoblast activity and decrease prostaglandin synthesis, similarly affecting callus formation at the docking site. Other factors causing delayed union include the patient's nutritional status, incomplete debridement, osteoporosis, and instability of the external fixator.

In addition to a reasonably designed external fixator, mastery of the principles of minimally invasive surgery, the radical debridement and keeping the bone ends flat can effectively reduce the incidence of delayed union. Acute shortening followed by distraction histogenesis have been shown to result in rapid and earlier healing of the docking site. However, this technique should not be used in patients with chronic infection and compromised soft tissue [36]. At present, the accordion technique and bone grafting are the main treatments for delayed union. Although accordion techniques can stimulate bone ends callus regeneration, their efficacy is often unpredictable, and there are no operating standards. In addition, the medullary canal can be reconstructed by drilling the bone ends through a wire. However, this operation should be limited to its specific indications to prevent the occurrence of bone infection. Early revision of the docking site was undertaken as soon as the transported segment had reached the target site, as recommended by many authors. We advocate early freshening of the fracture ends with removal of any interposed soft tissue after achieving docking. In our study, bone healing was achieved with compression with an external fixator after bone grafting in 20 patients due to factors of contact surface deviation. and the remaining patients achieved union after compression with an external fixator.

In the present study, we used the ASAMI scoring system to evaluate the effectiveness of the bone transport method. The rate of excellent and good bone and functional results was 95.1% and 90.3%, respectively. These results were similar to those of other studies.

Docking site nonunion and axial deviation or after-frame regenerate fracture were the major causes of failures. The main solutions for docking site complications focus on the following: (1) The accordion technique combined with minimally invasive percutaneous decortications to stimulate regeneration; (2) Among the modifications made in order to shorten the healing time at the docking site are grafting of the area, and plate and nail application at the site. (3) We recommend compression with an external fixator after bone grafting when docking site contact surface deviation. (4) Accurate study of the regenerate with radiographic methods and clinical tests to exclude premature removal of the external fixator and potential treatment failure.

The present study had several limitations. In this study, we performed a retrospective analysis of past cases and included fewer patients, which may have caused bias. This was a preliminary analysis of the treatment results, without a detailed discussion of the relevant influencing factors or a comparative analysis with other surgical methods. Further investigations, especially multi-centred trails with a larger sample size should be conducted to overcome the limitations of our study.

In conclusion, our review and the current evidence suggest that Ilizarov methods in the treatment of tibial bone defect resulted in satisfactory effects in bone results and functional results. Awareness of predictable complications favours prevention or early detection of anticipated complications that may improve the risk–benefit balance.

We believe experience has a great impact on the results of different procedures because follow-up and management of expected complications are cornerstones of treatment strategies. Future research should focus on reducing the difficulties associated with long bone transport, such as methods for enhancing the regeneration of bone. Advances through research to stimulate regeneration and reduce the duration of treatment will revolutionize limb lengthening surgery.

## Data Availability

The datasets analyzed during the current study are available from the corresponding author on reasonable request.

## References

[1] Goldstein Rachel Y, Jordan Charles J, McLaurin Toni M (2013). The evolution of the Ilizarov technique: part 2: the principles of distraction osteosynthesis. Bull Hosp Jt Dis.

[2] Masquelet AC, Begue T (2010). The concept of induced membrane for reconstruction of long bone defects. Orthop Clin North Am.

[3] Walker M, Sharareh B, Mitchell SA (2019). Masquelet reconstruction for posttraumatic segmental bone defects in the forearm. J Hand Surg Am.

[4] Omololu B, Ogunlade SO, Alonge TO (2002). Limb conservation using non vascularised fibular grafts. West Afr J Med.

[5] van Isacker T, Barbier O, Traore A (2011). Forearm reconstruction with bone allograft following tumor excision: a series of 10 patients with a mean follow-up of 10 years[J]. Orthop Traumatol Surg Res.

[6] Friedrich JB, Moran SL, Bishop AT (2008). Free vascularized fibular graft salvage of complications of long-bone allograft after tumor reconstruction. J Bone Joint Surg Am.

[7] Borzunov DY, Kolchin SN, Malkova TA (2020). Role of the Ilizarov non-free bone plasty in the management of long bone defects and nonunion: Problems solved and unsolved. World J Orthop..

[8] Rohilla R, Siwach K, Devgan A (2016). Outcome of distraction osteogenesis by ring fixator in infected, large bone defects of tibia[J]. J Clin Orthop Trauma.

[9] Veselý R, Procházka V (2016). Kalusdistrakce v léčení poúrazových defektů femuru a tibie [Callus Distraction in the Treatment of Post-Traumatic Defects of the Femur and Tibia]. Acta Chir Orthop Traumatol Cech.

[10] Mader JT, Cripps MW, Calhoun JH (1999). Adult posttraumatic osteomyelitis of the tibia. Clin Orthop Relat Res.

[11] Borzunov DY, Chevardin AV (2013). Ilizarov non-free bone plasty for extensive tibial defects. Int Orthop.

[12] Catagni MA, Azzam W, Guerreschi F (2019). Trifocal versus bifocal bone transport in treatment of long segmental tibial bone defects. Bone Joint J.

[13] Paley D (1990). Problems, obstacles, and complications of limb lengthening by the Ilizarov technique. Clin Orthop Relat Res.

[14] Paley D, Catagni MA, Argnani F (1989). Ilizarov treatment of tibial nonunions with bone loss. Clin Orthop Relat Res.

[15] Chimutengwende-Gordon M, Mbogo A, Khan W (2017). Limb reconstruction after traumatic bone loss. Injury.

[16] Borzunov D, Balaev P, Subramanyam K (2015). Reconstruction by bone transport after resection of benign tumors of tibia: a retrospective study of 38 patients. Indian J Orthop.

[17] Sala F, Thabet AM, Castelli F (2011). Bone transport for postinfectious segmental tibial bone defects with a combined Ilizarov/Taylor spatial frame technique. J Orthop Trauma.

[18] Giannoudis PV, Faour O, Goff T (2011). Masquelet technique for the treatment of bone defects: tips-tricks and future directions. Injury.

[19] Ilizarov GA (1989). The tension-stress effect on the genesis and growth of tissues: Part II. The influence of the rate and frequency of distraction. Clin Orthop Relat Res.

[20] Mekhail AO, Abraham E, Gruber B (2004). Bone transport in the management of posttraumatic bone defects in the lower extremity. J Trauma Injury Infect Crit Care.

[21] Ulrich S, Robert P, Jan F (2013). Clinical course, complication rate and outcome of segmental resection and distraction osteogenesis after chronic tibial osteitis. Injury.

[22] Xing T, Lei H, Shengsong Y (2013). Management of bone defects at tibial metaphysis by bone transport technique with linear-circular hybrid external fixators. Chin J Orthop Trauma.

[23] Trøite AG, Harald S, Per L (2005). In vivo assessment of regenerate axial stiffness in distraction osteogenesis. J Orthop Res.

[24] Behrens F, Searls K (1986). External fixation of the tibia. Basic concepts and prospective evaluation. J Bone Joint Surg Br.

[25] Giotakis N, Narayan B (2007). Stability with unilateral external fixation in the tibia. Strateg Trauma Limb Reconstr.

[26] Apivatthakakul T, Arpornchayanon O (2002). Minimally invasive plate osteosynthesis (MIPO) combined with distraction osteogenesis in the treatment of bone defects. A new technique of bone transport: a report of two cases. Injury.

[27] Emmanouil L, Mohamed K, Christian K (2011). Comparison of 39 post-traumatic tibia bone transports performed with and without the use of an intramedullary rod: the long-term outcomes. Int Orthop.

[28] Windhager R, Groszschmidt K, Tsuboyama T (1996). Recorticalization after bifocal internal bone transport in the double-plated sheep femur. J Orthop Res.

[29] Barbarossa V, Matković BR, Vucić N, Bielen M, Gluhinić M (2001). Treatment of osteomyelitis and infected non-union of the femur by a modified Ilizarov technique: follow-up study. Croat Med J.

[30] Meffert RH, Inoue N, Tis JE, Brug E, Chao EY (2000). Distraction osteogenesis after acute limb-shortening for segmental tibial defects. Comparison of a monofocal and a bifocal technique in rabbits. J Bone Joint Surg Am.

[31] Chen H, Teng X, Hu XH (2020). Application of a pre-filled tissue expander for preventing soft tissue incarceration during tibial distraction osteogenesis. World J Clin Cases.

[32] Yusufu A (2018). Hot spots of recent research in bone lengthening. China J Orthop Trauma.

[33] Lavini F, Dall'Oca C, Bartolozzi P (2010). Bone transport and compression-distraction in the treatment of bone loss of the lower limbs. Injury.

[34] Ziran B, Cheung S, Smith W (2005). Comparative efficacy of 2 different demineralized bone matrix allografts in treating long-bone nonunions in heavy tobacco smokers. Am J Orthop.

[35] Sen C, Erdem M, Gunes T (2007). Effects of diclofenac and tenoxicam on distraction osteogenesis. Arch Orthop Trauma Surg.

[36] Kevin T, Dror P, Cengiz S (2017). Bone transport versus acute shortening for the management of infected tibial non-unions with bone defects. Injury.

